# Piezo1 regulates autophagy in HT22 hippocampal neurons through the Ca^2+^/Calpain and Calcineurin/TFEB signaling pathways

**DOI:** 10.1371/journal.pone.0330282

**Published:** 2025-08-26

**Authors:** Yatong Wu, Yan Lu, Hao Zhang, Suhe Dong, Ziqing Zhang, Sinian Wang, Fengsheng Li, Shimin Yin

**Affiliations:** 1 The Postgraduate Training Base of Jinzhou Medical University (The PLA Rocket Force Characteristic Medical Center), Beijing, China; 2 Department of Neurology, The PLA Rocket Force Characteristic Medical Center, Beijing, China; 3 Department of Anesthesiology, The PLA Rocket Force Characteristic Medical Center, Beijing, China; 4 Department of Nuclear Radiation Injury and Monitoring, The PLA Rocket Force Characteristic Medical Center, Beijing, China; University College London, UNITED KINGDOM OF GREAT BRITAIN AND NORTHERN IRELAND

## Abstract

**Objective:**

To investigate the functional and molecular mechanisms by which Piezo1regulates HT-22 hippocampal neuronal autophagy, and to explore whether Piezo1 regulates hippocampal neuronal autophagy via the Ca^2+^/Calpain, CaMKKβ, or Calcineurin pathways.

**Methods:**

The impacts of Piezo1 inhibition, activation and gene knockdown on the autophagy of HT22 neurons was investigated by Western blotting, PCR and immunofluorescence. The changes of intracellular calcium (Ca^2+^) concentration were also observed. To pinpoint the specific downstream Ca^2+^ signaling pathway by which Piezo1 modulates autophagy, the calcium chelator BAPTA-AM, the Calpain inhibitor PD151746, and the CaMKKβ inhibitor STO609 were employed either alone or in combination.

**Results:**

Enhanced autophagy was observed when Piezo1 was activated using the agonist Yoda1, manifesting as increased release of autophagic vacuoles, enhanced LC3 II/LC3 I ratio, decreased p62 protein level, and elevated nuclear translocation and expression of the TFEB protein. ATG7 knockdown by ATG7 shRNA mitigated the effects of Yoda1 on LC3 II/LC3 I ratio and p62 protein levels. The Piezo1 inhibitor GsMTx4 partially reversed the autophagy caused by starvation in HT22 neurons while Yoda1 still activated autophagy in the presence of BDNF. Following Piezo1 knockdown, neuronal autophagy was decreased. Piezo1-induced autophagy was accompanied with an increased cytoplasmic concentration of Ca^2+^. The calcium chelator BAPTA-AM partly reversed Piezo1 activation-induced autophagy, which was also mitigated by blocking calcineurin/TFEB signaling or Calpain signaling.

**Conclusion:**

Piezo1 modulates the autophagy of HT-22 neurons by activating Ca^2+^/Calpain and Calcineurin/TFEB pathways.

## Introduction

Piezo1 is a mechanosensitive cation channel involved in regulating multiple pathophysiological functions. It has been found to control various forms of cell death, including apoptosis and ferroptosis [1,2]. For example, Piezo1-mediated Ca^2+^ overload can cause necrosis and apoptosis in pancreatic acinar cells [3]. Genetic deletion of Piezo1 is accompanied with compensatory increase in the activities of T-type voltage-gated Ca^2+^ channels, which results in p53-dependent aging and death phenotype of skeletal muscle stem cells [4]. Our previous research demonstrates that Piezo1 can modulate the process of apoptosis of neuron oxygen-glucose deprivation/reoxygenation damage via Ca^2+^/Calpain signaling [5]. Piezo1 can also regulate ferroptosis. Ionizing radiation enhances the degradation of E-cadherin and induces ferroptosis by upregulating the expression of Piezo1 protein in pulmonary endothelial cells [6]. Piezo1 activation is also found to induce ferroptosis via inhibition of GPX-4 in mouse chondrocytes [7]. Through the removal of harmful elements or modification of essential protein levels, cellular autophagy can modulate both apoptosis and ferroptosis [8]. However, it is unclear whether Piezo1 is involved in regulating autophagy.

Piezo1 channels regulate cytoplasmic calcium homeostatic balance and signaling mainly by mediating Ca^2+^ influx [9,10]. After treatment with the Piezo1 agonists Yoda1 or Jedi2, dorsal root ganglion neuronal Ca^2+^ transients were increased by at least one-fold, while the amount of Ca^2+^ transients was considerably decreased by the Piezo1 inhibitor GsMTx4 [11]. Although Piezo1 channels are primarily localized to the cell membrane, they can interact with and regulate the activity of sarco/endoplasmic reticulum Ca²⁺-ATPase (SERCA), prompting extracellular Ca² ⁺ influx and then facilitating the outflow of Ca^2+^ from the endoplasmic reticulum repertoire via inositol 1,4,5-trisphosphate (IP3) receptor (IP3R) type 2 (IP3R2)- cAMP signaling [12]. Furthermore, altered Ca^2+^ homeostasis is of the utmost importance in autophagy regulation. Research from Gordon *et al.* [13] revealed that Ca^2+^ is essential for protein cleavage by autophagic lysosomes. The initiation, nucleation, elongation, and closure of the isolation membrane (autophagosome precursor) are critical processes during the development of autophagosomes. Liquid-like focal adhesion kinase family interacting protein of 200 kD (FIP200) assembly can be induced by calcium transients on the ER surface and occurs through liquid-liquid phase separation. The FIP200 puncta then binds to the membrane VAPA/B and ATL2/3 for endoplasmic reticulum anchoring and acts as an autophagosome starting site for downstream autophagy proteins, leading to the development of autophagosome. The rapid Ca^2+^ chelating molecule BAPTA-AM blocks this action, while the slow Ca^2+^ chelator EGTA-AM does not work [14]. Transient receptor potential canonical (TRPC) channel TRPC6 activation induced Ca^2+^ influx increases autophagy levels in SH-SY5Y neuroblastoma cells [15]. In an acute pancreatitis model, the activation of Ca^2+^ ion channel Orai1 activates calcineurin and the transcription factor EB, which increases the expression of genes associated with autophagy [16]. In APP/PS1 double transgenic mice and SH-SY5Y cells, Aβ stimulates the production of autophagic vacuoles as well as the deposition of pathological autophagic vesicles through the receptor for advanced glycation end products (RAGE)/Ca^2+^ pathway. The aforementioned excessive autophagy is inhibited by the Ca^2+^ chelator BAPTA-AM [17]. However, it remains obscure whether Piezo1 controls neuronal autophagy via calcium signaling.

There are three main Ca^2+^ signaling pathways that have been found to regulate autophagy, which are calcineurin, CaMKKβ, and Calpain signaling. For example, Calpain and autophagy share intricate reciprocal regulation [18,19]. The CaMKKβ/AMPK pathway is reported to mediate autophagy triggered by Aβ treatment in SH-SY5Y cells [16]. Mitochondrial or metabolic stress can trigger mitochondrial autophagy through lysosomal Ca^2+^ release, followed by an increase in cytoplasmic Ca^2+^, and the activation of calcineurin, which binds to and dephosphorylates TFEB. The dephosphorylated TFEB then transfers to the nucleus and initiates autophagy-related gene expression [20,21]. Whether these signals contribute to the regulatory effects of Piezo1 on neuronal autophagy is still unknown. This study explores the specific signaling pathway through which Piezo1 regulates neuronal autophagy via Ca^2+^ signaling.

## Materials and methods

### Cell culture

Mouse hippocampal neuronal cell line (HT22) with short tandem repeats (STR) confirmation is purchased from Procell Life Science Technology (Wuhan, China). The cells were cultured in DMEM medium (Gibco, USA) with 10% fetal bovine serum (Gibco, USA), 100 U/mL penicillin, and 100 mg/mL streptomycin. The cells were subjected to incubation in a controlled environment with a temperature of 37°C, with saturated humidity and 5% CO_2_. The culture medium was changed every other day to ensure optimal growth and maintenance.

### Western blotting

Proteins were extracted from cultured neurons using RIPA lysis buffer (Thermo Scientific™, 78510) along with protease phosphatase inhibitors (NCM biotech, P002). The concentrations of protein were measured utilizing a BCA Protein Assay kit (Beyotime, China). Proteins were separated using 10–12% SDS-PAGE. Following wet transfer, 5% defatted milk in Tris Buffered saline Tween (TBST) was used for blocking for one hour. After that, the membranes were incubated with the primary antibodies (1:1000) at 4°C overnight. The following primary antibodies were employed: rabbit anti-PIEZO1 antibody (Cat#A4340, Abclonal), rabbit anti-LC3B (Cat#ab192890, Abcam), rabbit anti-SQSTM1/p62 (Cat#ab109012, Abcam), rabbit anti-TFEB (Cat# 13372–1-AP, Proteintech), rabbit anti-phosphorylated TFEB (Ser122) (p-TFEB, Cat#86843S, CST), rabbit anti- ATG7 (Cat#10088–2-AP, Proteintech) and mouse anti-GAPDH (internal reference, Cat# 60004–1-Ig, Proteintech). After three washing in a 1 x TBST solution, the membrane remained incubated for an hour at room temperature with a secondary antibody (Cat#SA00001–1; Cat#SA00001–2, Proteintech). The blots were visualized with SuperFemto ECL chemiluminescence Kit (Vazyme, Nanjing, China) and captured with a gel imaging system (Bio-Rad ChemiDoc™MP). The target bands’ density values were quantified using ImageJ software.

### Real-time reverse transcript quantitative PCR (RT-qPCR)

Total RNA was extracted from HT22 cells with TRIzol (Sigma, United States). The PrimeScript™ RT reagent kit was used to reverse transcribe the mRNA to cDNA, and a PCR instrument (Bio-Rad) was used to amplify the cDNA. Lastly, the Bio-Rad CFX Manager software was used to extract the raw results. The 2^-ΔΔ^Ct method was employed to analyze the results, with GAPDH serving as the internal control. The following primer sequences were employed: PIEZO1, Forward: 5′-CGGACAGTGAGGAGGAAGAGGAG-3′ and Reverse: 5′-CCTGTTCACGACGACGCTGCCTTAG-3′; p62, Forward: 5′-GGATGGGGACTTGGTTGC-3′ and Reverse: 5′-TCACAGATCACATTGGGGTGC-3′; TFEB, Forward: 5′-AAGTTCGGGAGTATCTGTCTG-3′ and Reverse: 5′-GGTTGGAGCTGATATGTAGCA-3′, and GAPDH, Forward: 5′-GGTTGTCTCCTGCGACTTCA-3′ and Reverse: 5′- TGGTCCAGGGTTTCTTACTCC-3′.

### Gene knockdown by siRNA

RIBOBIO (Guangzhou, China) designed and manufactured siRNAs that specifically targeted PIEZO1(Cat#siG171016103537, RIBOBIO) and TFEB (Cat#siG14516154705, RIBOBIO). The target sequences for Piezo1 siRNA1 were 5’-TCGGCGCTTGCTAGAACTTCA-3’ [22–26]. We designed three TFEB-targeting siRNAs: TFEB-siRNA1 (target sequence, 5’-CCAAGAAGGATCTGGACTT-3’), TFEB-siRNA2 (target sequence, 5’-GACTCAGAAGCGAGAGCTA-3’), and TFEB-siRNA3 (target sequence, 5’-GCCTGGAGATGACTAACAA-3’). When the neuronal cells had reached 70% confluency, 5 µL of Lipofectamine 3000 (Thermo Fisher Scientific, USA) was combined with 250 µL of Opti-MEM (Gibco, USA) in a sterile tube, mixed gently, and incubated at room temperature for 8–10 minutes. Following this, siRNA (50 or 100 nM; RIBOBIO) [27] or corresponding negative control (NC) siRNA (sequence proprietary per the manufacturer’s policy of RIBOBIO) was diluted in 250 µL of Opti-MEM, added to the pre-prepared Lipofectamine 3000-Opti-MEM mixture, and incubated for an additional 20 minutes at room temperature to form transfection complexes. The complexes were then evenly transferred to the cell-containing wells, and the cells were incubated for 48–72 hours with media refreshed thereafter to remove residual transfection reagents. The efficiency of the transfection and gene knockdown was verified by Western blotting and RT-qPCR.

### Lentiviral infection

Lentiviral vectors for ATG7 knockdown were designed by and purchased from GeneChem (Shanghai, China). Three short hairpin RNAs (shRNAs) targeting mouse ATG7 were designed: ATG7-shRNA1 (target sequence: 5‘-TTCCTGAGAGCATCCCTCTAA-3’), ATG7-shRNA2 (target sequence: 5’-CAGCCTCTGTATGAATTTGAA-3’), and ATG7-shRNA3 (target sequence: 5’-ACCCAAAGTTCTTGATCAGTA-3’). A negative control (NC) shRNA (sequence: 5’-TTCTCC GAACGTGTCACGT-3’) was also used. HT22 cells were seeded into 6-well plates at a density of 4 × 10⁴ cells per well. When cell confluence reached approximately 30%, 30 μL of lentivirus (1 × 10⁸ TU/ml) and Hitrans polybrene (25 × stock) were added to the complete DMEM medium (multiplicity of infection, MOI = 20). After 12–16 hours of incubation, the medium was replaced with fresh complete medium. Cell status was monitored at 24–72 hours post-infection. Lentiviral infection efficiency was first assessed at 72 hours. Cells were then selected for stable infection by incubation in medium containing 2 μg/ml puromycin for 48 hours. ATG7 expression was verified by Western blotting and RT-qPCR to evaluate knockdown efficiency.

### Immunofluorescence

Before treatment, cells were incubated in a glass-bottom cell culture dish (Cat#801022, NEST). When 50% to 70% confluency was observed, HT-22 cells were fixed for 30 minutes in 4% ice-cold paraformaldehyde. For cell permeabilization, 0.2% Triton X-100 was used. After blocking with 10% goat serum blocking solution (Cat# ZLI-9056, ORIGENE) for 1 hour at room temperature, the samples were then exposed to the following antibodies: mouse anti-LC3B antibody (1:200, Cat# 83506S, CST), rabbit anti-SQSTM1/p62 (1:200, Cat# ab109012, Abcam), and rabbit anti-TFEB (1:200, Cat# 13372–1-AP, Proteintech) at 4°C overnight. After incubation with fluorescently labeled goat anti-mouse IgG H&L (AF594) (1:400, Cat#550042, ZEN-BIOSCIENCE) or goat anti-rabbit IgG H&L (AF488) (1:400, Cat#550037, ZEN-BIOSCIENCE) for 1 hour at room temperature, DAPI (C1002, Beyotime, China) was further incubated for 5–6 minutes. An inverted fluorescence microscope (Leica, Germany) was utilized to capture the immunofluorescence staining. According to the guidelines for the use and interpretation of assays for monitoring autophagy published by Daniel Klionsky and colleagues [28], puncta of LC3 and p62 in the cells can also serve as marker for autophagosome. The average LC3B and p62 puncta number were quantified and presented as puncta number/cell. We calculated the puncta number and mean value of LC3B and p62, and the number of autophagosomes in the cell using ImageJ separately. Detection of the fluorescence ratio of TFEB protein in the nucleus and cytoplasm was used to calculate the TFEB nuclear/cytoplasmic ratio.

### Detection of autophagic vacuoles

Autophagic vacuoles were detected with the Autophagy Assay Kit (Cat# ab139484, Abcam) according to the procedures from the manufacturer. After drug treatment, cells were washed with 1 × assay buffer, then incubated with Hoechst 33342 Nuclear Stain and green detection reagent which will show bright fluorescence upon incorporation into pre-autophagosomes, autophagosomes, and autolysosomes (autophagolysosomes) for 30 minutes at 37°C in the dark. Following incubation, cells were rinsed with 1 × assay buffer to remove unbound reagent and observed under a fluorescent microscope (Leica, Germany).

### Fluorescent Ca^2+^ imaging

The intracellular Ca^2+^ levels were measured using Fluo4-AM (Dojindo, Kumamoto, Japan). Following treatment, HT22 cells were incubated with Fluo4-AM (5 µM) at 37°C for 45 minutes. After washing, the Fluo4-AM fluorescence signal at 516 nm (excited at 494 nm) was observed using a fluorescent microscope (Leica, Germany).

### Statistical analyses

Statistical analysis and plotting were performed using GraphPad software 8 Trial version. The data was expressed as the mean ± standard error of means (SEM). One-way Analysis of Variance (ANOVA) was conducted for comparison, and a Tukey test was used for post hoc pairwise comparison. Unless otherwise stated, every experiment was conducted thrice independently. p < 0.05 (two-tailed) implied a statistically significant difference.

## Results

### Piezo1 regulates HT22 neuronal autophagy

**Fig 1:**
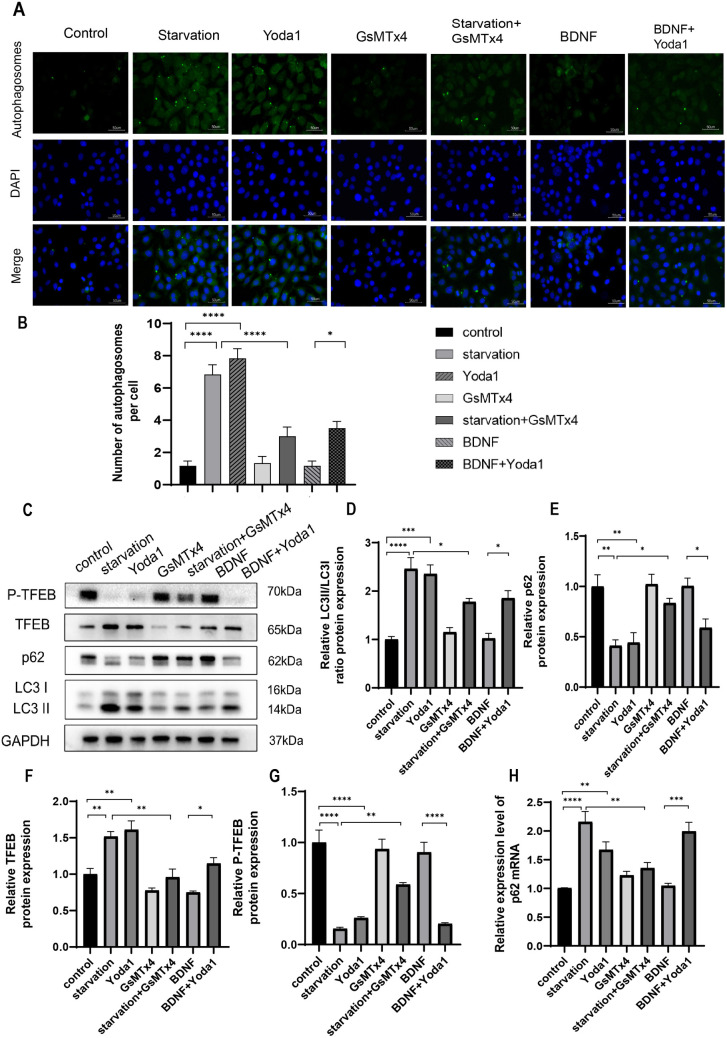
Piezo1 modulates autophagy of HT22 neurons. **(A)** Autophagy measured using The Autophagy Assay Kit and represented as the number of autophagic vacuoles. Scale bar: 50µm. **(B)** Statistical analysis of the number of autophagosomes (APs) per cell. Data were expressed as mean ± SEM (n = 6) and analyzed by one-way ANOVA with Tukey’s test. **(C-G)** Representative Western blotting strips **(C)** and statistical histograms **(D-G)** showing the effects of Piezo1 activation (Yoda1) or inhibition (GsMTx4) on the expression of autophagy-associated proteins. Piezo1 agonist Yoda1 (3µM) and starvation increase LC3 II/LC3 I ratio **(D)**, decreases p62 protein levels **(E)**, elevate TFEB protein levels **(F)**, and decrease TFEB phosphorylation levels **(G)**. The Piezo1 inhibitor GsMTx4 (5µM) reverses starvation-induced autophagy. Yoda1 still induces autophagy in the presence of BDNF. **(H)** Yoda1 elevates the expression of p62 mRNA. Western blot experiments were performed in three independent samples. Values were expressed as mean ± SEM. * p < 0.05; ** p < 0.01; *** p < 0.001; and **** p < 0.0001.

**Fig 2:**
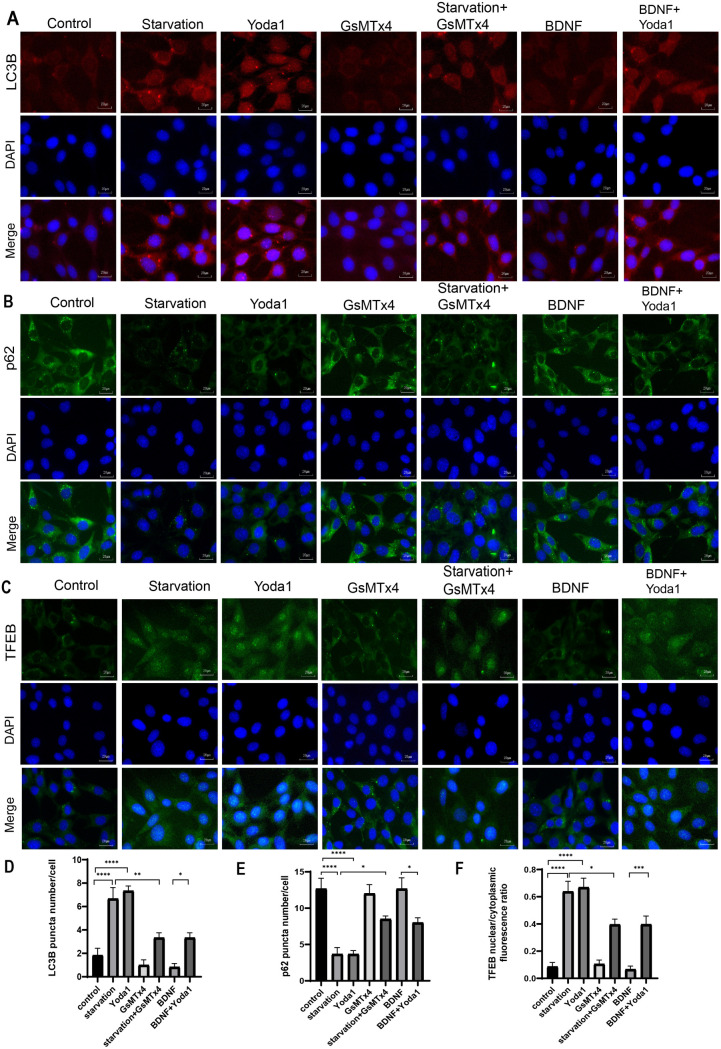
Piezo1 modulates autophagy of HT22 neurons as supported by immunofluorescence. Immunofluorescence confirming the effect of Piezo1 activation or inhibition on changes of autophagy-related protein LC3B **(A)** and p62 **(B)**, as well as TFEB nuclear translocation **(C)** in HT22 neurons. Cell nuclei were counter-stained with DAPI (blue). Scale bar: 20µm. **(D-E)** The average LC3B and p62 puncta number were quantified and presented as puncta number/cell. Data were expressed as mean ± SEM (n = 6) **(F)** The bar graph shows the mean ± SEM of TFEB nuclear/cytoplasmic ratios from six independent samples (one-way ANOVA with Tukey’s post-hoc test). * p < 0.05; ** p < 0.01; *** p < 0.001, and **** p < 0.0001.

**Fig 3:**
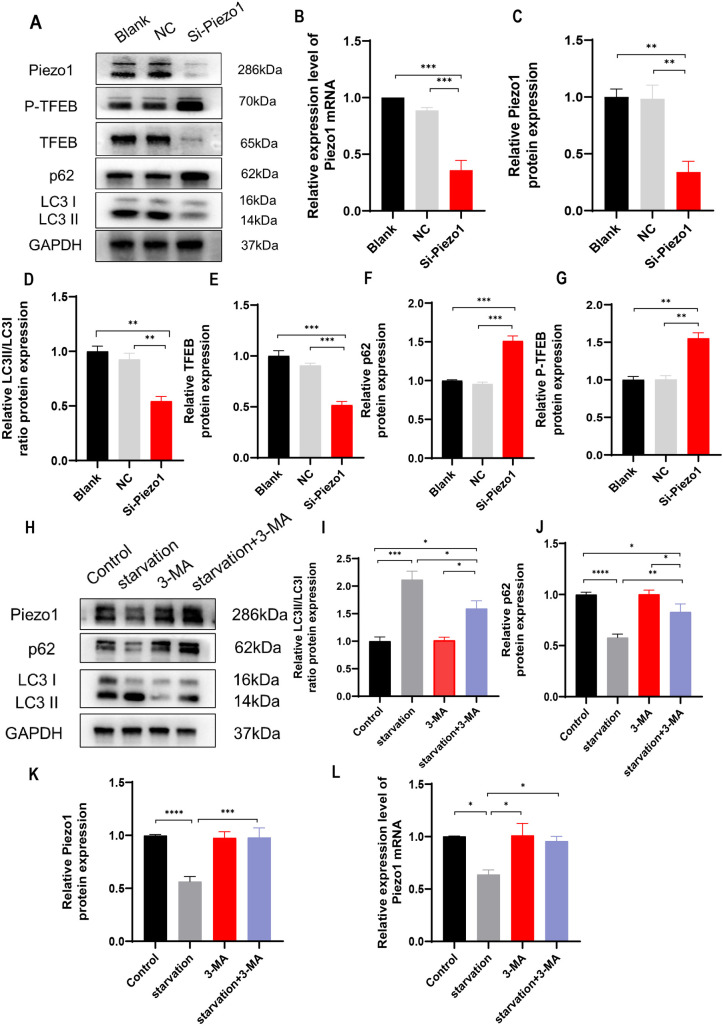
Inhibition of Piezo1 signaling suppresses autophagy of hippocampal neurons. **(A-G)** Changes of the expression of autophagy-related proteins shown in representative Western blotting blot strips **(A)** and statistical histograms **(C-G)** after Piezo1 knockdown using 100 nM of Piezo1-specific siRNA **(A and B)**. **(H-L)** Changes of Piezo1 protein (**H and K**) and mRNA (**L**) expression after starvation or 3-MA (a specific autophagy inhibitor) treatment. Western blot experiments were performed in three independent samples. Values are expressed as mean ± SEM. * p < 0.05; ** p < 0.01; *** p < 0.001, and **** p < 0.0001.

**Fig 4:**
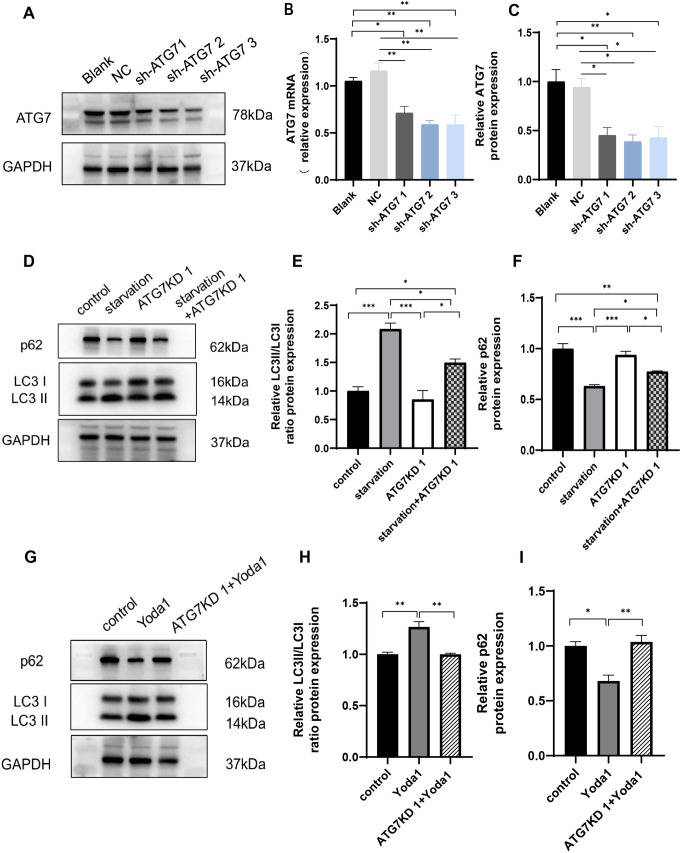
ATG7 knockdown mitigates the effects of Piezo1 activation on LC3 II/LC3 I ratio and p62 protein levels. **(A-C)** Western blotting analysis (**A and C**) and qPCR (**B**) showing successful knockdown of ATG7 mRNA and protein in HT22 cells. **(D-F)** ATG7 knockdown blocked starvation-induced increase in LC3 II/LC3 I ratio (**D and E**) and decreased p62 protein (**D and F**) levels in HT22 cells. **(G-I)** Knockdown of ATG7 reversed Yoda1-induced increase in LC3 II/LC3 I ratio (**G and I**) and decreased p62 protein (**H and I**) levels. Western blot experiments were performed in three independent samples. Values were expressed as mean ± SEM. * p < 0.05; ** p < 0.01; *** p < 0.001.

We first examined the impact of Piezo1 activation, inhibition, or knockdown on autophagy of HT22 cells. The experiment comprised seven groups: control (CON), starvation, Yoda1 (selective Piezo1 activator [29], 3 μM; Cat#HY-18723, MCE), GsMTx4 (Piezo1 inhibitor, not completely specific, but currently the only available [30], 2.5 μM; Cat#P1205, Selleck), starvation + GsMTx4 (5 μM), BDNF (50 ng/ml; Cat#Z03208, GenScript), and BDNF (50 ng/ml) + Yoda1 (3 μM). Control cells were cultured in Dulbecco’s Modified Eagle Medium (DMEM) (Gibco, USA) with 10% fetal bovine serum and subjected to the same vehicle treatment duration as experimental groups. Autophagy was induced by starving HT-22 neurons for six hours in a glucose-free DMEM without serum addition (Cat#PM150270, Procell) [31], which served as a positive control. Exogenous BDNF acts as a negative control to inhibit neuronal autophagy [32]. In comparison with control, treatment with the Piezo1 activator Yoda1 dramatically increased the number of autophagic vacuoles (Fig 1A and 1B). p62 protein levels and LC3 conversion from LC3 I to LC3 II are commonly used as autophagy markers [33,34]. The results revealed that Yoda1 treatment led to elevated LC3 II/LC3 I ratio, decreased p62 protein levels as well as increased TFEB protein levels, and reduced TFEB phosphorylation (Fig 1C–1G). Immunofluorescence demonstrated increased number of LC3 puncta (Fig 2A and 2D), decreased p62 puncta number and p62 fluorescence intensity (Fig 2B and 2E), and increased TFEB nuclear/cytoplasmic fluorescence ratio (Fig 2C and 2F), indicating augmented autophagy. Conversely, the use of GsMTx4, a Piezo1 inhibitor, in neurons undergoing starvation-induced autophagy results in a decrease in the total amount of autophagic vacuoles (Fig 1A and 1B), a reduction in the LC3 II/LC3 I protein ratio and TFEB protein levels (Fig 1D and 1F), and an increase in p62 and P-TFEB protein levels (Fig 1E and 1G). BDNF inhibits neuronal autophagy [35]. When applied after BDNF treatment, Yoda1 still increased the number of autophagic vacuoles (Fig 1A and 1B), elevated the LC3 II/LC3 I ratio, enhanced TFEB protein levels, and decreased p62 and P-TFEB protein levels (Fig 1C–1G). PCR results exhibited increased expression of p62 mRNA with Yoda1 treatment (Fig 1H), with GsMTx4 reversing the increase in p62 mRNA expression. Yoda1 also increased p62 mRNA expression in BDNF-treated neurons (Fig 1H). Furthermore, to confirm Piezo1 dependency, we supplemented GsMTx4 experiments with Piezo1 knockdown using specific siRNA. Piezo1 siRNA, blank control, and negative control NC were added into the culture media 48 hours before cell collection. The results showed that Piezo1 knockdown significantly reduced TFEB and LC3 II/LC3 I ratio, while increasing p62 and P-TFEB levels (Fig 3D–3G, supplementary Fig S2 in S1 File), indicating the inhibition of autophagy. Notably, in starved HT22 neurons, Piezo1 protein expression was reduced. 3-Methyladenine (3-MA) is a widely used inhibitor of autophagy and autophagosome formation. We added the inhibitor 3-MA to starvation-treated cells, and found 3-MA decreased the LC3 II/LC3 I ratio, increased p62 protein levels, and increased the expression of Piezo1 (Fig 3H–3L). The expression of ATG7, an enzyme responsible for the lipidation of LC3 I to LC3 II was further suppressed by lentiviral transfection of ATG7 shRNA to interrupt autophagy [36]. The effects of starvation and Piezo1 activation on LC3 and p62 was then observed in these autophagy-deficient cells. When autophagy was disrupted by ATG7 knockdown (Fig 4A–4C), the effects of starvation (Fig 4D–4F, supplementary Fig S3 in S1 File) and Yoda1(Fig 4G–4I, supplementary Fig S3 in S1 File) on LC3 II/LC3 I ratio and p62 protein levels were greatly mitigated. Taken together, these findings support that Piezo1 signaling regulates autophagy in hippocampal neurons.

### The regulation of autophagy by Piezo1 relies on Ca^2+^ signaling

**Fig 5:**
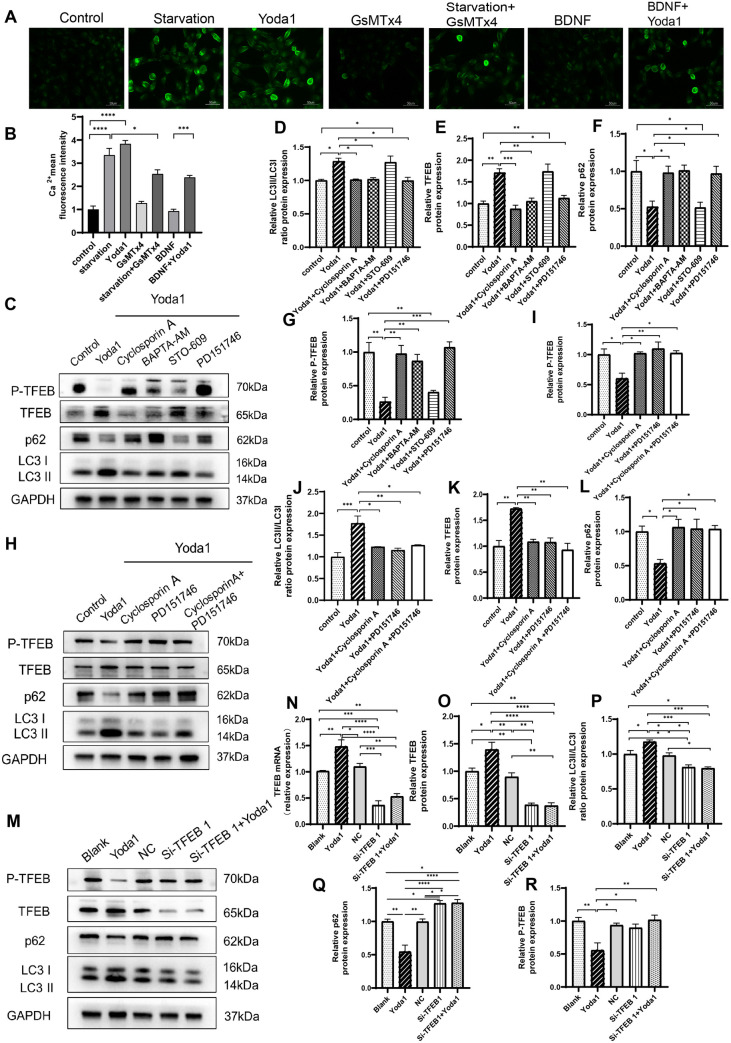
Piezo1 regulates autophagy through Ca^2^^+^/Calpain and Calcineurin/TFEB pathways. **(A)** Intracellular Ca^2+^ concentrations measured using Fluo4-AM. **(B)** Histograms showing differences in Ca^2+^ concentrations across groups. **(C-G)** Screening for the specific Ca^2+^ signaling pathways through which Piezo1 regulates autophagy. Following Yoda1 (2.5µM) treatment, rapid calcium chelator BAPTA-AM (10µM), Calpain inhibitor PD151746 (20µM), Calcineurin inhibitor Cyclosporin A (1µM), or the CaMKKβ inhibitor STO609 (10µM) was added. Changes in LC3 **(C and D)**, TFEB **(C and E)**, and p62 (**C and F**) protein contents, and TFEB phosphorylation (**C and G**) in HT-22 neurons were examined. Yoda1-induced autophagy in neuronal cells was partially inhibited by BAPTA-AM, PD151746, and Cyclosporin **A. (H-L)** Potentially synergistic inhibitory effects of Cyclosporin A and PD151746 on Yoda1-induced autophagy measured by Western botting. **(M-R)** Effects of TFEB knockdown using 100 nM of TFEB-specific siRNA on Yoda1-induced autophagy. Histograms and Western blotting images demonstrate that TFEB knockdown partially reverses Yoda1-induced activation of autophagy. Western blot experiments were performed in three independent samples. Values were expressed as mean ± SEM. * p < 0.05; ** p < 0.01; *** p < 0.001; and **** p < 0.0001.

Piezo1 is reported to act mainly through Ca^2+^ signaling [23]. Whether the autophagy-regulating effect of Piezo1 was dependent on Ca^2+^ signaling was examined. We found that both starvation and Yoda1 increased intracellular Ca^2+^ concentration in HT22 neurons, while GsMTx4 reduced the starvation-induced rise of Ca^2+^ concentration (Fig 5A and 5B). When Ca^2+^ was chelated using BAPTA-AM (10µM), Western blotting results TFEB protein levels and LC3 II/LC3 I ratio were significantly decreased by BAPTA-AM compared to the Yoda1 treatment-alone group (Fig 5C–5E), which were accompanied by increased p62 protein and P-TFEB levels (Fig 5F and 5G). These findings suggest that Piezo1 induces autophagy through Ca^2+^ signaling.

### Piezo1 regulates autophagy through Ca^2+^/Calpain and Calcineurin/TFEB pathways

**Fig 6:**
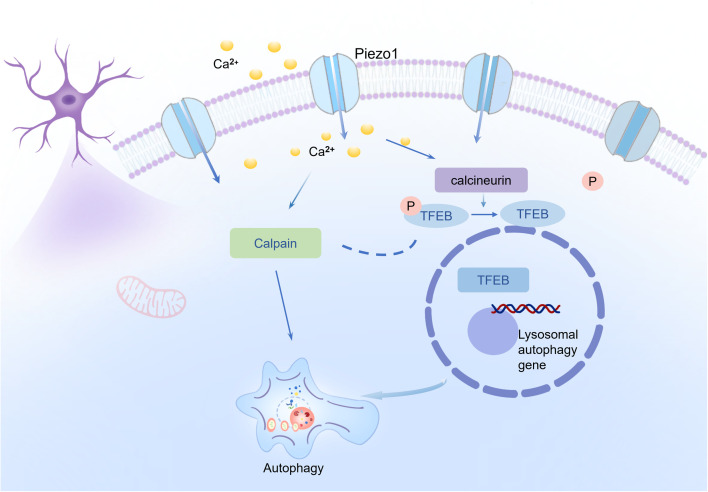
A graphic summary of the mechanism by which Piezo1regulates HT22 neuronal autophagy. Piezo1 facilitates Ca^2+^ influx, which induces autophagy of HT-22 cells by activation of Calpain and Calcineurin/TFEB pathways.

Autophagy can be regulated by Ca^2+^ via the actions of Calpain, Calcineurin, and CaMKKβ signaling [37]. The specific Ca^2+^ downstream signaling pathways, including calcineurin/TFEB, Calpain, and CaMKKβ, that mediate the impact of Piezo1 on autophagy was identified. There were six groups for this set of experiment: control (CON), Yoda1 (3 µM), Yoda1 + BAPTA-AM (Ca^2+^ chelator, 10µM; Cat#S7534, Selleck), Yoda1 + Cyclosporin A (calcineurin inhibitor [38], 1µM; Cat#M1831, AbMole), Yoda1 + STO609 (CaMKKβ inhibitor [39], 10µM; Cat#S8274, Selleck), Yoda1 + PD151746 (Calpain inhibitor [10,40], 20µM; Cat#HY-19749, MCE). We found that the Calpain inhibitor PD151746 and the Calcineurin inhibitor Cyclosporin A significantly decreased LC3 II/LC3 I ratio (Fig 5C and 5D), inhibited TFEB expression (Fig 5C and 5E), increased p62 protein level (Fig 5C and 5F), and increased TFEB phosphorylation (Fig 5C and 5G) in Yoda1 treated cells. In contrast, the CaMKKβ inhibitor STO609 showed no such effect. To explore whether TFEB knockdown could mitigate the autophagy-inducing effect of Piezo1 activation, 100 nM of TFEB siRNA was added to the culture media 48 hours before Yoda1 stimulation. When TFEB expression was silenced using siRNA, the Yoda1’s autophagy-promoting action was also mitigated (Fig 5M–5R, supplementary Fig S1 in S1 File). Combined use of PD151746 with Cyclosporin A revealed no synergistic effects on autophagy inhibition (Fig 5H–5L). Taken together, these results suggest that Piezo1 regulates neuronal autophagy through Ca^2+^/Calpain and Calcineurin/TFEB pathways (Fig 6).

## Discussion

In the present study, we discovered that activation of Piezo1 induces neuronal autophagy, while the Piezo1 antagonist GsMTx4 inhibits starvation-induced autophagy. Regarding signaling pathways, Piezo1 acts via the Ca^2+^/Calpain and Calcineurin/TFEB pathway to modulate HT22 cell autophagy. The main findings are summarized in Fig 6.

Piezo1 is expressed in both neurons and glial cells [41]. Our recent research found an enriched environment reduces the negative effects of sleep deprivation on fear memory by modifying the Piezo1/Calpain/autophagy pathway in the basal forebrain [42], without clarification of which cell types are involved. A previous study reports that transcranial magnetic stimulation activates Piezo1 to enhance microglial autophagy, thereby promoting the phagocytosis and degradation of β-amyloid, and attenuating AD-associated synaptic plasticity impairments [43]. When oxidized low-density lipoprotein (ox-LDL) is administered to human umbilical vein endothelial cells (HUVECs), Piezo1 knockdown reverses the elevated levels of autophagy [44]. These results suggest that Piezo1 activation may induce autophagy. However, it has also been documented that Piezo1 causes apoptosis in human myeloid nucleus pulposus (NP) cells by inhibiting autophagy under excessive mechanical stress [2]. Therefore, Piezo1’s regulatory impact on autophagy might be cell-specific. In this study, we found that Piezo1 activation enhances autophagy in HT22 neurons. Moreover, autophagy triggered by starvation was decreased following the administration of the Piezo1 inhibitor GsMTx4 (Fig 1). After Piezo1 was knocked down by siRNA, neuronal autophagy was also decreased. The use of 3-MA, an inhibitor of autophagy, ameliorated starvation-induced neuronal autophagy, resulting in decreased expression of Piezo1 (Fig 3). ATG7 knockdown abolished starvation- and Yoda1-induced LC3 II production and decreased p62 protein levels (Fig 4). These findings indicate that Piezo1 signaling is crucial for the autophagy of neurons.

The order of cation selectivity of Piezo1 is Ca^2+^ > K^+^ > Na^+^> Mg^2+^ [45]. Research revealed that the Piezo1 activator Yoda1 increases extracellular Ca^2+^ influx and facilitates the release of calcium from intracellular calcium reservoirs through transient receptor potential (TRP) cation channels in astrocytes [46]. The fast Ca^2+^ chelator BAPTA-AM effectively prevents free Ca^2+^ from diffusing away from the plasma membrane entrance point, but not the slow chelator EGTA [45]. Syeda *et al.* [47] found that EGTA cannot abolish the Ca^2+^ responses after Yoda1 stimulation in HEK293T cells. The current study showed that BAPTA-AM significantly reduces Ca^2+^ influx, with reversed autophagy triggered by Piezo1 activation (Fig 5). Besides Ca^2+^, other cations such as Mg^2+^ [48] and K^+^ [49] have been reported to modulate autophagy. However, we did not detect the changes in these ions or their potential mediating roles in Piezo1-induced autophagy.

Ca^2+^ has been found to exert a regulatory function on the process of autophagy through several signaling pathways, including Calpain, Calcineurin, and CaMKKβ [37]. It has also been discovered in earlier research that Piezo1 regulates the aforementioned pathways. For example, neuronal oxygen-glucose deprivation/reoxygenation injury is shown to be mitigated by inhibition of Piezo1’s stimulation of Ca^2+^/Calpain signaling [5] and endothelial tip cell branching extension or retraction during cerebral vascular development [50]. Cyclic mechanical stretch (CMS) could induce annulus fibrosus cells (AFCs) apoptosis by Piezo1/Ca^2+^/Calpain-2/Caspase-3 pathway [51]. Conditional deletion of Piezo1 attenuated the effects of isoproterenol-induced myocardial hypertrophy in mice by reducing Ca^2+^ overload and Calpain activity. The cardiac hypertrophy induced by Piezo1 activation was mediated by Ca^2+^-related calcineurin and Calpain activation [52,53]. We examined whether the above signaling pathways were involved in regulating Piezo1-induced autophagy. It was discovered that Yoda1-induced neuronal autophagy is partially inhibited by the Calpain inhibitor PD151746 and the calcineurin inhibitor CsA (Fig 5). In contrast, autophagy was not significantly altered by the CaMKKβ inhibitor STO609 in Yoda1-treated cells (Fig 5). Furthermore, it was found that the reduced expression of TFEB by siRNA reversed the neuron autophagy-promoting effect induced by Yoda1 (Fig 5). These findings support that Piezo1 modulates neuronal autophagy probably by Calpain and Calcineurin/TFEB signaling.

We further found there is no synergistic effect between PD151746 and Cyclosporin A, suggesting that the two may act via upstream and downstream regulations. Research has shown that calcineurin and Calpain interact in a way that allows Calpain to cleave calcineurin and then increase its phosphatase activity [54,55]. However, we did not investigate the potential relationships further. Another limitation is being a crucial transcriptional regulator of lysosomal formation, TFEB stimulates the expression of several genes related to autophagy and lysosomal biogenesis, including LAMP1、CTSD、CTSL、UVRAG、SQSTM1、MAPLC3B、ATG9 and others [56], which was not examined in the current study. GsMTx-4 mainly targets mechanosensitive channels from the Piezo and transient receptor potential (TRP) families, indicating GsMTx-4 is not specific, or even selective, for Piezo1 [57], despite the fact that GsMTx-4 is the only inhibitor available. Moreover, when high concentrations of siRNA are used during experiments, the risk of off-target effects increases. Therefore, we employed at least two distinct siRNAs targeting the same gene or utilized validated siRNA sequences.

In conclusion, we found that Piezo1 activation triggers neuronal autophagy through Ca^2+^/Calpain and Calcineurin/TFEB. Considering that dysfunctional autophagy has been demonstrated to be an essential contributor to the onset and course of some neurodegenerative diseases [58], the regulation of neuronal autophagy through Piezo1 signaling has the potential to be a therapeutic strategy for the treatment of neurodegenerative diseases.

## Supporting information

S1 File Supplementary Figure. (PDF)

S2 File Raw images. Original immunofluorescence images for Fig 2 and Fig5.
(PDF)

S3 File Raw images. Original image of blot for Fig 1 and Fig3–5.
(PDF)

S1 Data Dataset. (XLSX)
