# Supplementary material for: Piezo1 regulates autophagy in HT22 hippocampal neurons through the Ca2+/Calpain and Calcineurin/TFEB signaling pathways
Source: PLoS One. 2025 Aug 26;20(8):e0330282. doi: 10.1371/journal.pone.0330282 (PMC12380351; doi:10.1371/journal.pone.0330282)
Supplement: S1 File — (PDF) [file pone.0330282.s001.pdf]

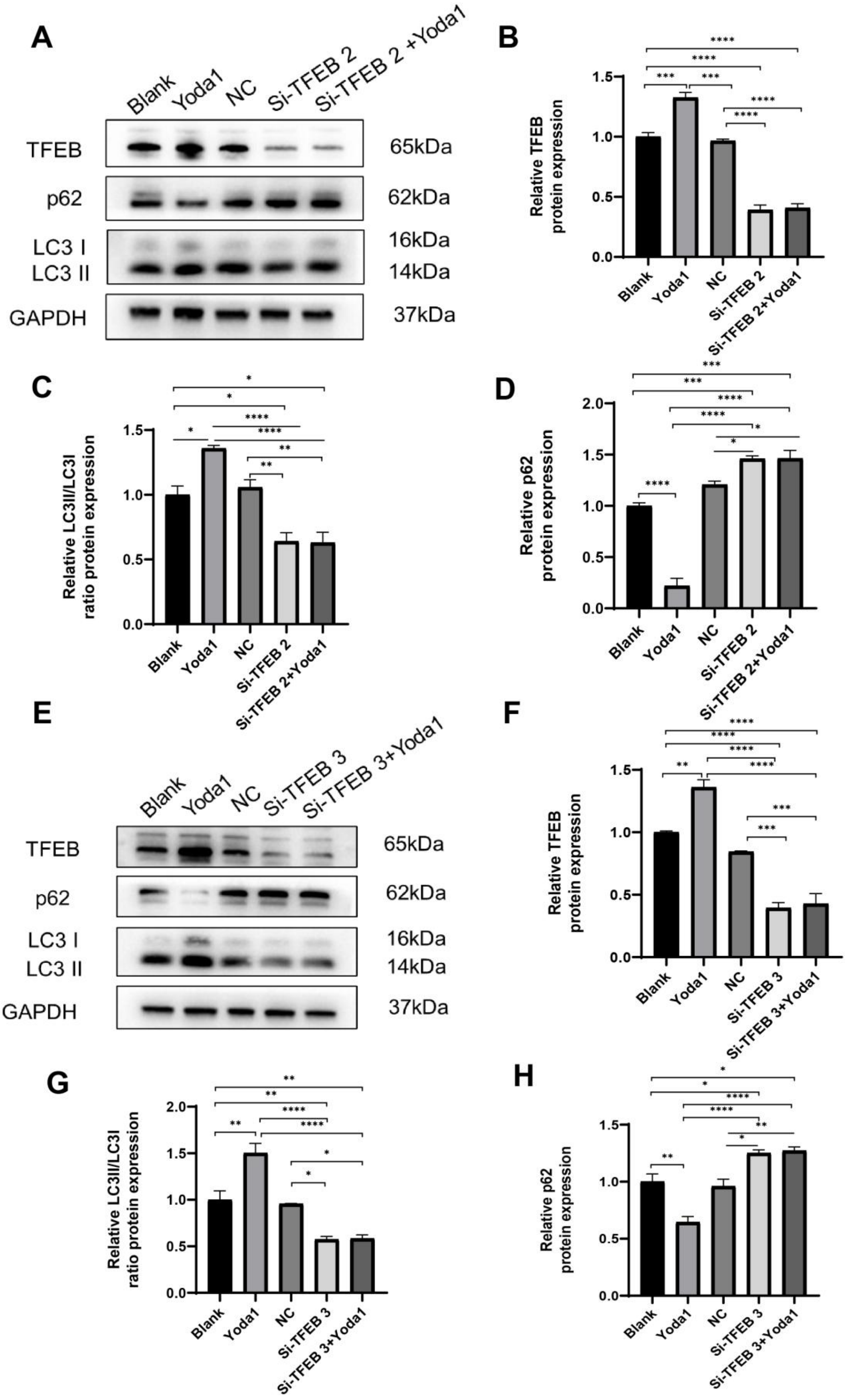
A
Blank
Yoda1
NC
Si-TFEB 2
Si-TFEB 2 +Yoda1
TFEB
65kDa
p62
62kDa
LC3 I
16kDa
LC3 II
14kDa
GAPDH
37kDa
B
Relative TFEB protein expression
C
Relative LC3II/LC3I ratio protein expression
D
Relative p62 protein expression
Si-TFEB 2+Yoda1
E
Si-TFEB 3
Si-TFEB 3+Yoda1
F
G
H

Supplementary Fig. S1. TFEB knockdown mitigated Yoda1-induced autophagy. To minimize off-target effects, three distinct siRNA sequences (si-TFEB 1-3) were employed for TFEB silencing. Western blotting analysis (**A and E**) and quantitative densitometry (**B and F**) demonstrate that si-TFEB 2 and si-TFEB 3 achieved significant suppression of TFEB protein expression compared to non-targeting control (**NC**). Western blotting analysis shows that TFEB knockdown by si-TFEB 2 **(A-D)** and si-TFEB 3 **(E-H)** partly reversed Yoda1-induced increase in LC3 II/LC3 I ratio and decrease in p62 protein level. * $p < 0.05$; ** $p < 0.01$; *** $p < 0.001$; and **** $p < 0.0001$.

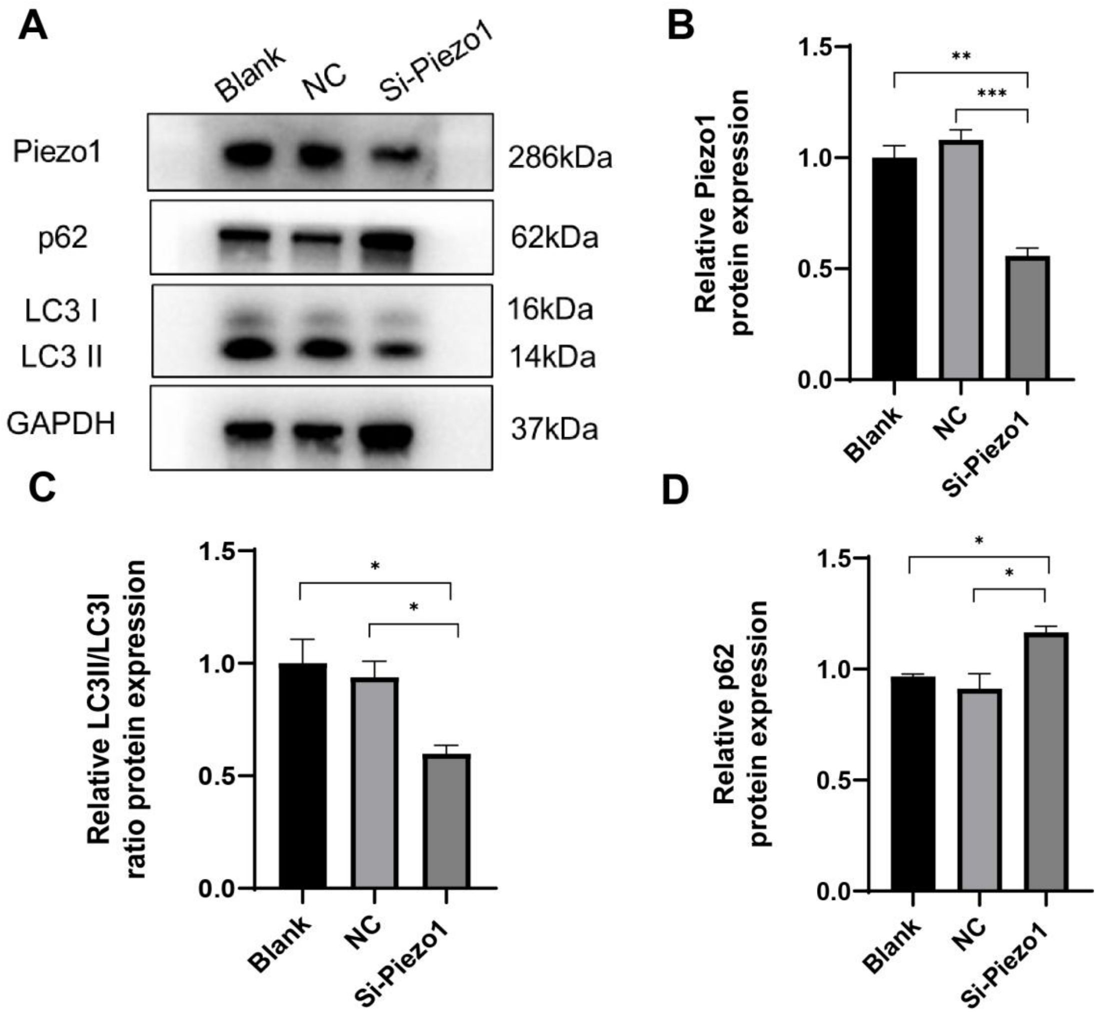

Supplementary Fig. S2. Piezo1 knockdown inhibited neuronal autophagy. A concentration of 50 nM of Piezo1-specific siRNA was used for Piezo1 silencing. Western blot analysis **(A)** and statistical histograms **(B-D)** showed that si-Piezo1 (50 nM) inhibited the expression of some piezo1 proteins compared with the control and NC groups. Western blot analysis **(A-D)** showed that si-Piezo1 (50 nM) resulted in a partial decrease in the LC3 II/LC3 I ratio and an increase in the p62 protein level. The knockdown efficiency using a concentration of si-Piezo1 (50 nM) was significantly lower than the knockdown at 100 nM (see Fig. 2). * $p < 0.05$; ** $p < 0.01$; *** $p < 0.001$; and **** $p < 0.0001$.

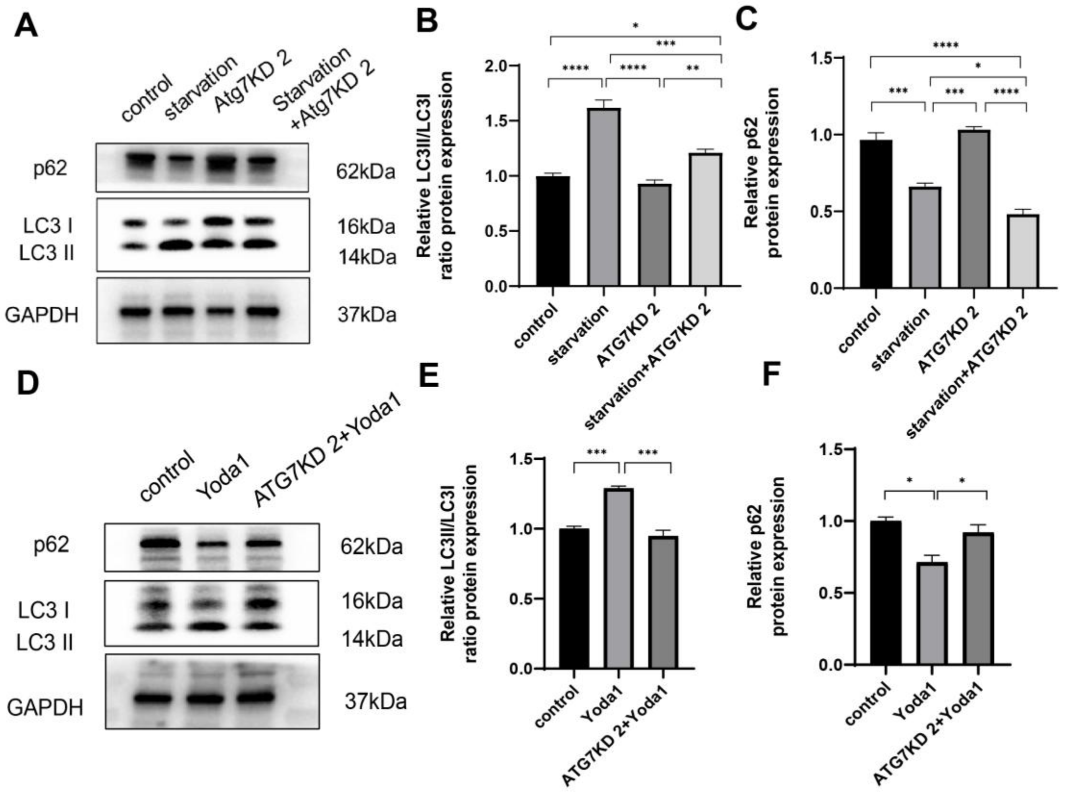

Supplementary Fig. 3. ATG7 knockdown attenuated starvation and Yoda1-induced autophagy. To reduce off-target effects, two different shRNA sequences (shRNA1-2) were used in experiments to examine the effect of ATG7 silencing on autophagy. Western blot analysis **(A and D)** and statistical histograms **(B-F)** showed that ATG7KD 2 **(A-F)** reversed the starvation and Yoda1-induced increase in LC3 II/LC3 I ratio and decrease in p62 protein levels. * $p < 0.05$; ** $p < 0.01$; *** $p < 0.001$; and **** $p < 0.0001$.
