# Supplementary material for: Piezo1 regulates autophagy in HT22 hippocampal neurons through the Ca2+/Calpain and Calcineurin/TFEB signaling pathways
Source: PLoS One. 2025 Aug 26;20(8):e0330282. doi: 10.1371/journal.pone.0330282 (PMC12380351; doi:10.1371/journal.pone.0330282)
Supplement: S2 File — Original immunofluorescence images for Fig 2 and Fig5. (PDF) [file pone.0330282.s002.pdf]

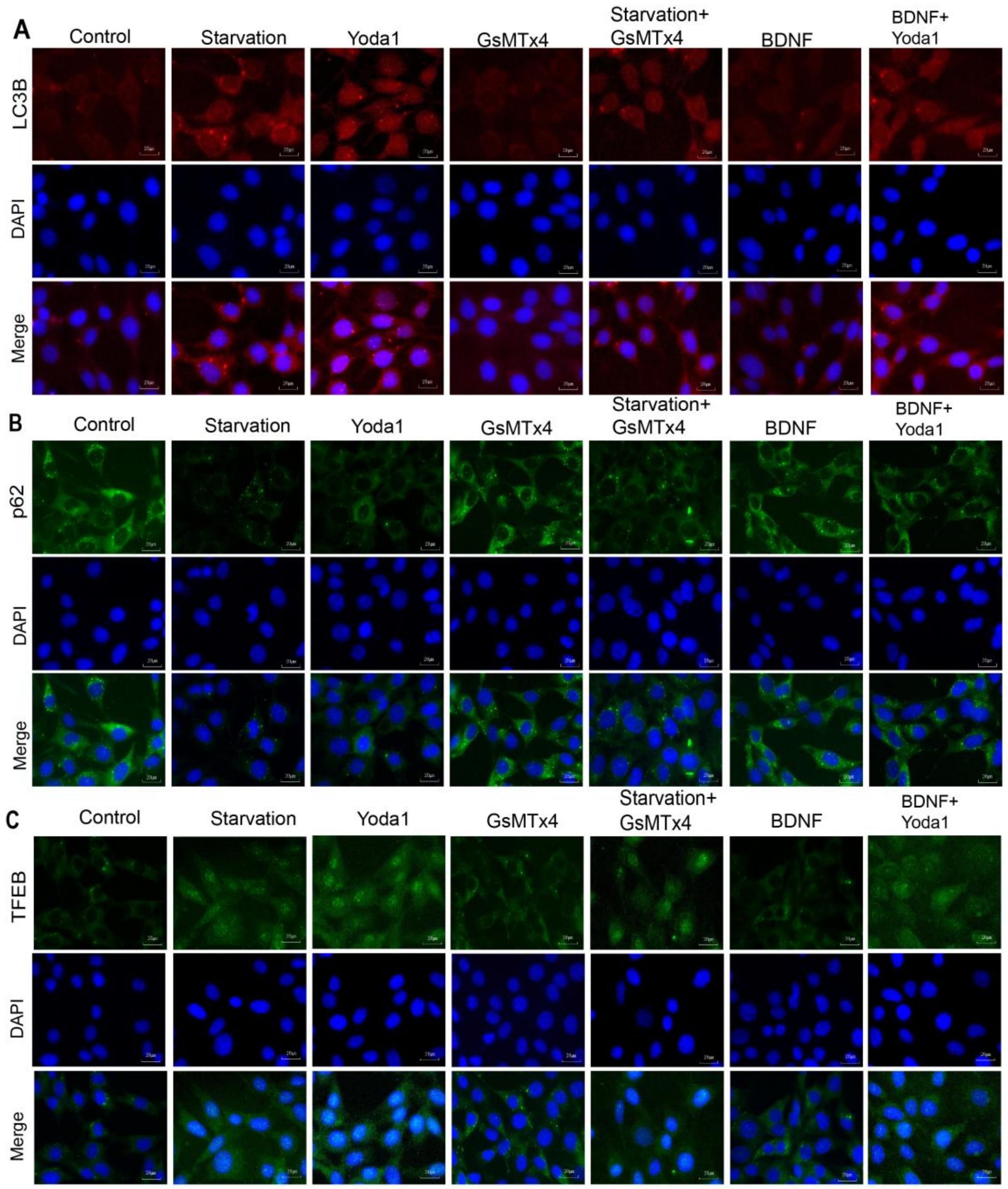

Raw images.Immunofluorescence confirming the effect of Piezo1 activation or inhibition on changes of autophagy-related protein LC3B (A) and P62 (B), as well as TFEB nuclear translocation (C) in HT22 neurons. Cell nuclei were counter-stained with DAPI (blue). Scale bar: 20μm.
