# Supplementary figures and images for: Piezo1 regulates autophagy in HT22 hippocampal neurons through the Ca2+/Calpain and Calcineurin/TFEB signaling pathways
Source: PLoS One. 2025 Aug 26;20(8):e0330282. doi: 10.1371/journal.pone.0330282 (PMC12380351; doi:10.1371/journal.pone.0330282)

Figure1-C

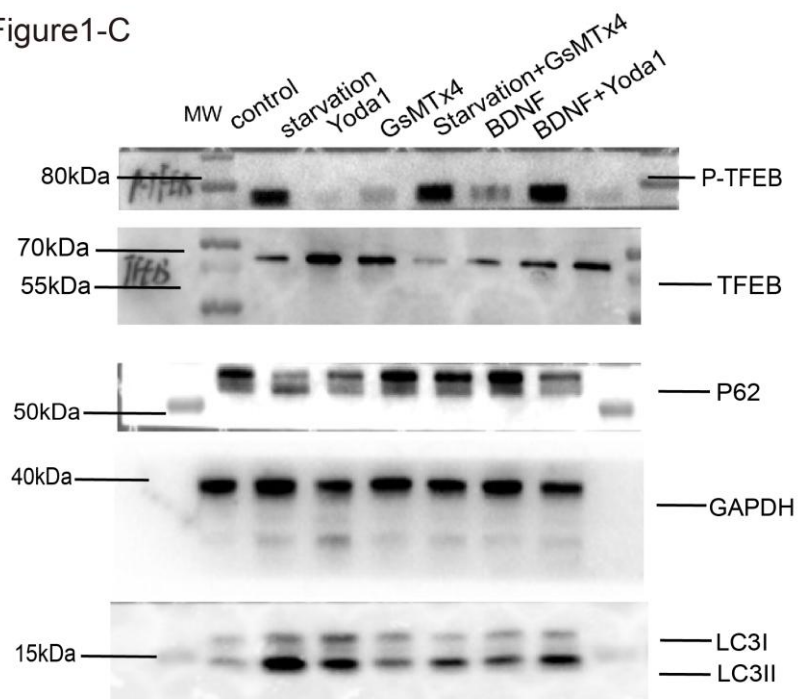

Figure3-A

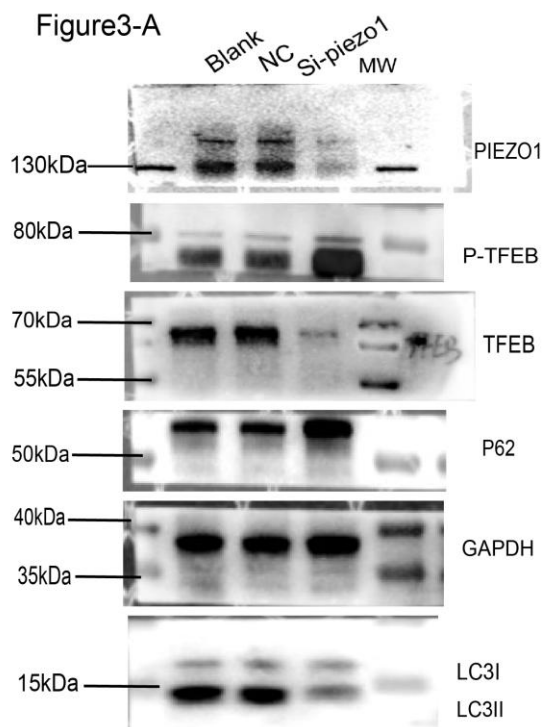

F3-H

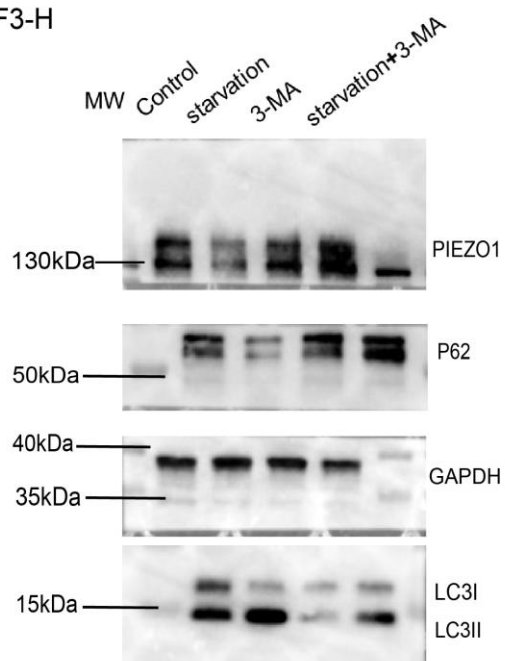

Figure4

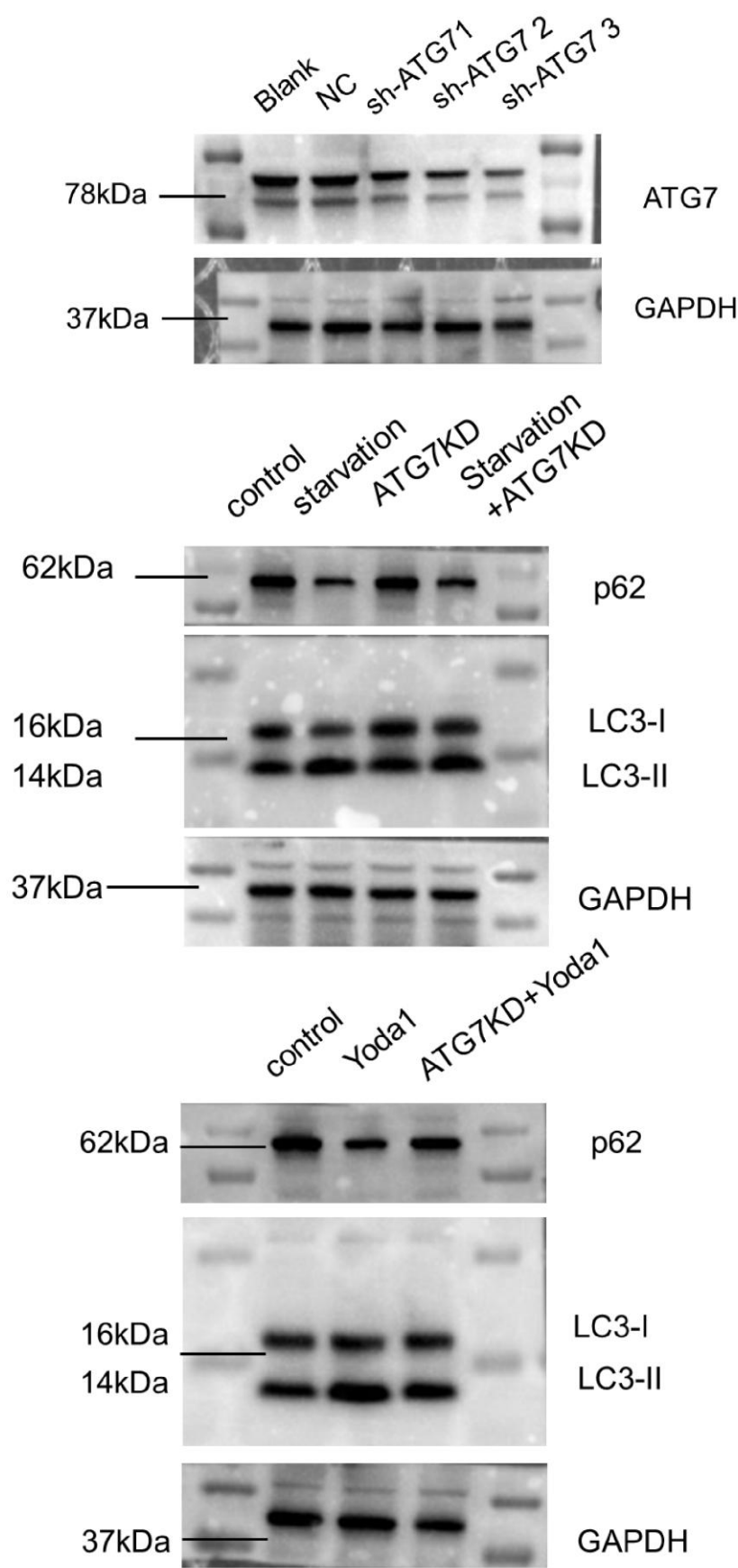

Figure5

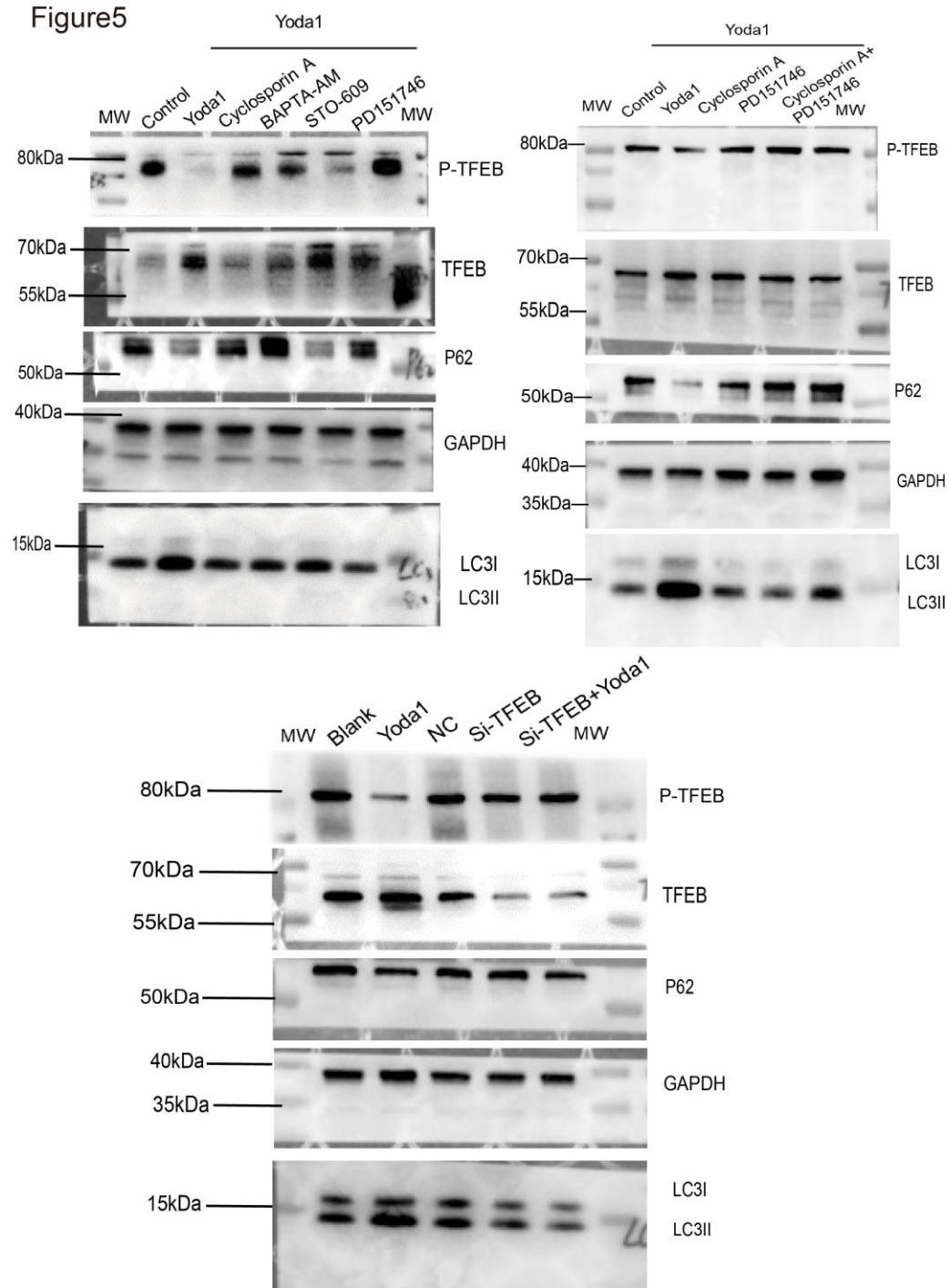

Supplement: S3 File — Original image of blot for Fig 1 and Fig3–5. (PDF) [file pone.0330282.s003.pdf]
